# Clinic of Professor Dunglison

**Published:** 1845-01

**Authors:** Samuel G. White

**Affiliations:** Georgia


### CLINICAL LECTURES AND REPORTS.

### PHILADELPHIA HOSPITAL.

*Saturday, December* 7, 1844.

### CLINIC OF PROFESSOR DUNGLISON.

Reported by Mr. Samuel G. White, of Georgia.

Professor Dunglison commenced the lecture by observing that before he took up the further consideration of the diseases of the respiratory organs, on which he was engaged at the last clinical lecture, he would draw the attention of the class to a case at present in the wards, on which he should make a few pathological and therapeutical observations. It was one of

RHEUMATISM, not of that violent inflammatory character to which the term acute arthritis has been more particularly applied, but rather a subacute form of the disease.

The patient, a middle aged man, has suffered for several years from an intermitting inflammation of the wrist joint, which recently has become more permanent, and gives him much uneasiness. In all such cases, it is well to direct attention to the condition of the heart, which is peculiarly liable to become implicated in acute, and subacute rheumatic affections. When this viscus becomes affected, it may be indicated by the presence of a *bruit de soufflet*, or bellows sound, produced by a narrowing of the orifice of the aorta, and especially from inflammation of the endocardium, and the consequent effusion of lymph, which impedes more or less the passage of the blood through the organ. In the present instance, however, the sounds of the heart are perfectly normal, and we infer there is no inflammation of the endocardium. On entering the hospital, the patient, who was of dissipated habits, and had been much exposed to the weather, complained of pain in the articulations; his pulse was frequent and indicative of inflammatory excitement, and there were slight tremors, such as usually accompany delirium tremens. From the condition of the pulse, it was considered necessary to have recourse to venesection, when the blood exhibited a thick buffy coat, with a very firm clot and cupped appearance, indicating a manifest augmentation of the fibrinous element. [The professor here exhibited the blood to the class, which presented in a marked manner the modifications just alluded to.]

Although the buffy coat is generally considered indicative of inflammatory action, it is very important to remember, that so far from being always characteristic of that condition, it may occur in states of the system the very opposite to the inflammatory; thus, it is very often present in chlorosis, as the clinical class had an opportunity some time since of witnessing. There is, however, a manifest difference in these cases in the consistence of the clot, being very firm in inflammation, but loosely coherent in chlorosis. It must, also, be borne in mind, that the "*bruit de soufflet*," which is supposed to be characteristic of endocarditis, may also occur where no such inflammation exists, but where there is a predominance of the watery portions of the blood, as in chlorosis. In these cases, the sound seems due to the impulse of thin fluid in its passage through the cavities of the heart.

In the patient before the class, there is considerable erethism of the nervous system, conjoined with the inflammatory symptoms.

The professor considers the inflammation of acute rheumatism to be peculiar, and certainly not identical with ordinary acute internal inflammation, as in pneumonia. In rheumatic affections, venesection is rarely resorted to with the same degree of confidence as it is in common inflammatory diseases. Indeed, considerable discrepancy of opinion exists among practitioners as to the propriety of bloodletting. Some regarding the disease to be decidedly inflammatory, as indicated by the state of the pulse and appearance of the blood, believe an active antiphlogistic treatment to be most required. Others entertain an opposite opinion, thinking that active depletion is not only unnecessary, but may be positively injurious by favoring the metastasis of the inflammation to an internal and vital organ. There are certainly cases, sometimes met with in private practice, and in the majority of those seen in hospitals, in which the active use of the lancet could not prove otherwise than injurious. In the one under consideration, where there is a complication with nervous erethism, especial caution is demanded in the farther use of depleting measures. The professor observed, that he had always considered rheumatism to be, like erysipelas, in part neuropathic, and frequently to demand a very different treatment from that which would be required in active internal inflammation. Tonics, as the sulphate of quinia, which act by the impression they make on the nervous system, are often, indeed, the best remedies that can be employed. The view as to its mixed inflammatory and neuropathic character derives support, not only from the effect of remedies, but from the extreme changeableness of the phlegmasia,—the appearances of inflammation shifting their seat in a few hours, or disappearing altogether without leaving the slightest organic alteration: they would seem indeed, to be more analogous to hyperæmia, than to true inflammation. It is of much importance to distinguish between these two conditions. In the former, there is simply an augmentation of the quantity of blood circulating in the part, which by over distending the vessels may render the circulation through them tardy, and occasion, also, nervous distension: it is, in fact, the stage that precedes true inflammation, and if it continue sufficiently long may terminate in it. When, however, positive inflammation has taken place, organic changes are produced in the parts, attended with effusion and an alteration of the natural secretions. Effusion also occurs in rheumatism, but it appears to be little more than the simple transudation of the liquor sanguinis from the overloaded vessels; hence the evidences of inflammation are exceedingly fugitive in acute rheumatism, and pass rapidly from one joint to another. From these and other facts, we are warranted perhaps in the conclusion, that this affection is of a mixed neuropathic and inflammatory character.

Granting this to be the pathology of acute rheumatism, we are led to prescribe a plan of treatment essentially different from that demanded in true inflammation; and hence we can comprehend that tonics may not be as injurious as might be supposed, if we regarded it differently.

The lecturer's experience leads him to place much confidence in the use of the sulphate of quinia in the treatment of certain forms and stages of rheumatism. He has witnessed the antiphlogistic and the tonic treatment practised exclusively in certain hospitals, and he is bound to say, that he has never seen bad effects from the latter. Some, indeed, consider the bark to be as effective in acute rheumatism as it is in intermittent fever.

From the immense doses of sulphate of quinia given in Paris, symptoms of cerebral excitement, and of general nervous erethism have been produced, but these effects do not follow its use in smaller quantities; and if it be granted, that when given to the extent just mentioned it may have been injurious, in less quantity it may be, and often is, productive of much benefit. In the majority of cases, however, the lecturer considers the mixed plan of treatment most effective, and he would recommend the disease to be treated in its early stages antiphlogistically;—abstracting blood, when necessary, generally or topically; administering large doses of the nitrate of potassa,—as advised in the last century, by Dr. Brocklesby, and now revived,—giving colchicum, which is a decided acro-narcotic, and consequently proper in mixed neuropathic and inflammatory conditions; and, later on in the disease, endeavouring to modify the neuropathic condition by the use of tonics—as the sulphate of quinia. This mode of treatment will be pursued in the present instance, and its results will be reported on a future occasion.

Acute rheumatism, like some other affections, seems to run a fixed course, usually continuing five or six weeks, although its duration may be occasionally shortened by properly directed measures.

The professor then introduced a female, affected with

THORACIC DISEASE, which served to further illustrate certain points connected with his lectures at the College on the physiology of respiration. The patient, a female, complains of difficulty of breathing and cough, which has continued since last June, and which she refers to exposure to wet. She now has burning of the feet and hands towards evening, with occasional, though not profuse, sweating; coughs more in the morning on rising than at any other period of the day, and expectorates a considerable quantity of frothy mucus.

Inspection of the chest indicates a slight diminution in the elevation of the right, compared with the left side, and percussion under the clavicle of the same side affords a dull sound. The main phenomenon developed by auscultation, is a great increase in the expiratory murmur—a sign, the value of which, in the diagnosis of pulmonary tuberculosis was first pointed out by Dr. James Jackson, Jr., of Boston. The pulsation of the heart, in this case, may be heard quite distinctly on the right side, which, as can be readily comprehended, may be owing to consolidation of the lung,—the sound being more readily conveyed through a solid organ. Posteriorly, the expiratory murmur is very audible; and on the left posterior part of the chest, a mucous rhonchus is distinguishable. The diagnosis may be stated to be consolidation with softening.

The treatment, in these hospital cases, is generally altogether revellent, consisting in making counter-irritation over the chest, with the use of eutrophics—as the iodide of potassium; but it need scarcely be said, that little benefit can be expected from these remedies,—the tubercles generally going on to softening, uninfluenced by any treatment.

The attention of the class was in conclusion directed to some interesting

PATHOLOGICAL SPECIMENS.

The first of these was from the patient Hannah S., aged 60, who, it will be recollected, laboured under hæmoptysis. (*Examiner*, Nov. 30, 1844, p. 278.) She continued to expectorate considerable quantities of blood until death, which appeared to be produced by asphyxia, from the sudden discharge of fluid from a cavity in the lung, which was not, however, blood, but puriform. The bronchial tubes were found filled with this matter on examination after death. During life, it seemed probable that the blood expectorated was poured out from an ulcerated vessel; but, on examination after death, it was impossible to determine whether this were really the case. Certainly, the ulcerated vessel, if it existed, did not open into the cavity, although it might open elsewhere. The lower part of the lung was engorged with blood, and it was at first supposed that at this point the hemorrhage might have taken place; but the most attentive examination could detect no open vessel; and, besides, this congested state of the lower portion of the lung is very commonly met with in those who do not die of hæmoptysis; hence nothing certain can be deduced from this appearance.

There is great difficulty in determining the true pathological cause of the hemorrhage in this case. It may have been the result of simple transudation; or an ulcerated vessel may exist at some point which has not been detected. Farther examination will be made to determine this point. The lungs do not contain so much tubercular deposition as is frequently met with in such cases. It is often, indeed, found that old persons dying from tuberculosis do not present such extensive disease of the lungs as those at an earlier age. In the young individual, both lungs are generally more or less affected, but, in this case, only the right seems diseased, and only a portion of this. The lecturer here took occasion to point out the physiological arrangement of the fibrous structure of the bronchia, which was well exhibited in the specimen.

The liver of this patient also presented—what is not very common—a deposition of tubercular matter in its parenchyma, of the presence of which no indication existed during life. Tubercular matter, deposited in this viscus, may be either similar to that found in the lungs—strumous—or it may be scirrhous. In the present instance, the tubercles are isolated and softened, and do not make the creaking noise which is characteristic of scirrhus when the knife is drawn through it.

The kidneys presented nothing abnormous, except that in one of them there was an exudation of blood into its substance, constituting what has been called "*renal apoplexy.*" There were a few ossific depositions, as often occur in advanced life, in the aorta, near the sinuses of Valsalva. The heart, generally, was healthy. It is to the impediment offered by these depositions to the passage of the blood along the aorta, that the "saw sound" and "rasp sound," heard during the systole and diastole of the heart, are often referable, rather than to ossification of the semilunar valves, as the lecturer has noticed in several instances.

The next case was exceedingly interesting from the number of pathological lesions existing at the same time, which rendered it surprising that the individual should have lived so long. The specimens were from the man referred to at the last lecture, who was considered to be affected with gangrene of the lung. The patient entered the house with every indication of a broken down constitution, and, for important reasons, was not subjected to much physical examination. As then stated, he was presumed to have gangrene of the lung, although some uncertainty had existed in the diagnosis, as the fœtor of the breath might possibly have been due to ptyalism. On making a necroscopical investigation, no portion of the lung was found especially fetid: they were engorged, as often happens, when any cardiac affection exists which obstructs the return of the blood. Slight consolidation was present at the summit of the right lung, but this was not to any amount. The heart was very much enlarged, and exceedingly flabby and fatty; the pericardium was roughened with a thin albuminoid secretion—the result of inflammatory action. The professor here remarked, that there were two results of pericarditis—one in which there was an effusion of solid matter, by which it becomes adherent to the surface of the heart, and glues the pericardium to it, impeding its motions; the other characterized by a liquid effusion, constituting hydrops pericardii. He also observed, that cases are not unfrequently met with, in which an attack of pericarditis has run its course without being indicated by any marked phenomena; and he instanced one that occurred in his private practice, where it was extremely difficult to form a positive diagnosis, in consequence of bronchitis coexisting, although it was suspected, and proved to be present by examination after death. Wherever, he said, much difficulty of breathing exists, without other signs of pulmonary mischief, pericarditis should be suspected. In the present case, the heart was not only large and flabby, but the chordæ tendineæ were very small, with an insufficiency on the part of the valves to close the enlarged auriculo-ventricular openings, especially on the right side. On the semilunar valves of the aorta a very large vegetation existed, with some cartilaginous degeneration of the valves themselves, which no doubt gave no inconsiderable impediment to the passage of the blood, and, doubtless, permitted aortic regurgitation. The lecturer took occasion to allude to the great importance to the class of an attentive examination of such pathological results; to enable them to deduce the influence such conditions must exert on the physiological actions of the parts immediately concerned, and the constitutional disturbance resulting from them.

The liver was also very much enlarged, and presented a granulated appearance, owing to hypertrophy of the biliary element of the organ, producing the first stage of what has been termed "cirrhosis of the liver." Notwithstanding the condition of the liver, there was, during life, no manifestation of dropsy.

The spleen presented nothing peculiar, except that it was somewhat larger, more flabby, and softer than usual; but there was an interesting pathological condition of the kidneys, which consisted in the great disproportion in their volume; the right being greatly hypertrophied, and the left extremely small, and lobated. There was a considerable amount of adipous matter deposited near their pelves, but nothing, in other respects, abnormous,—the small kidney appearing as healthy as the other; and it no doubt performed its functions to a certain extent healthily. From the great difference in their size, it is probable that the hypertrophy of the right kidney originated from its having the principal part of the function to execute, although it may be a question whether it was not a congenital conformation.

The great interest of this case, as before stated, is in the circumstance, that the patient should have survived so long with such serious alterations in so many important organs.

